# Use of Commonly Available Technologies for Diabetes Information and Self-Management Among Adolescents With Type 1 Diabetes and Their Parents: A Web-Based Survey Study

**DOI:** 10.2196/ijmr.4504

**Published:** 2015-12-29

**Authors:** Sarah E Vaala, Korey K Hood, Lori Laffel, Yaa A Kumah-Crystal, Cindy K Lybarger, Shelagh A Mulvaney

**Affiliations:** ^1^ Vanderbilt University School of Nursing Nashville, TN United States; ^2^ Stanford University Department of Pediatrics Palo Alto, CA United States; ^3^ Joslin Diabetes Center Boston, MA United States; ^4^ Vanderbilt University Medical Center Department of Biomedical Informatics Nashville, TN United States; ^5^ Vanderbilt University Medical Center Department of Pediatrics Nashville, TN United States

**Keywords:** diabetes mellitus, Type 1, adolescent, technology, adoption, self-management, self-care

## Abstract

**Background:**

For individuals with Type 1 diabetes (T1D), following a complicated daily medical regimen is critical to maintaining optimal health. Adolescents in particular struggle with regimen adherence. Commonly available technologies (eg, diabetes websites, apps) can provide diabetes-related support, yet little is known about how many adolescents with T1D use them, why they are used, or relationships between use and self-management.

**Objective:**

This study examined adolescent and parent use of 5 commonly available technologies for diabetes, including proportions who use each technology, frequency of use, and number of different technologies used for diabetes. Analyses also investigated the reasons adolescents reported for using or not using technologies for diabetes, and factors correlated with adolescents’ technology use. Finally, this study examined relationships between the type and number of technologies adolescents use for diabetes and their self-management and glycemic control.

**Methods:**

Adolescents (12-17 years) and their parents (N=174 pairs), recruited from a pediatric diabetes clinic (n=134) and the Children with Diabetes community website (n=40), participated in this Web-based survey study. Glycosylated hemoglobin (A1C) values were obtained from medical records for pediatric clinic patients. Adolescents reported their use of 5 commonly available technologies for diabetes (ie, social networking, diabetes websites, mobile diabetes apps, text messaging, and glucometer/insulin pump software), reasons for use, and self-management behavior (Self-Care Inventory-Revised, SCI-R).

**Results:**

Most adolescents and parents used at least one of the 5 technologies for diabetes. Among adolescents, the most commonly used technology for diabetes was text messaging (53%), and the least commonly used was diabetes websites (25%). Most adolescents who used diabetes apps, text messaging, or pump/glucometer software did so more frequently (≥2 times per week), compared to social networking and website use (≤1 time per week). The demographic, clinical, and parent-technology use factors related to adolescents’ technology use varied by technology. Adolescents who used social networking, websites, or pump/glucometer software for diabetes had better self-management behavior (SCI-R scores: beta=.18, *P*=.02; beta=.15, *P*=.046; beta=.15, *P*=.04, respectively), as did those who used several technologies for diabetes (beta=.23, *P*=.003). However, use of diabetes websites was related to poorer glycemic control (A1C: beta=.18, *P*=.01).

**Conclusions:**

Adolescents with T1D may be drawn to different technologies for different purposes, as individual technologies likely offer differing forms of support for diabetes self-management (eg, tracking blood glucose or aiding problem solving). Findings suggest that technologies that are especially useful for adolescents’ diabetes problem solving may be particularly beneficial for their self-management. Additional research should examine relationships between the nature of technology use and adolescents’ T1D self-management over time.

## Introduction

In the United States, approximately 20,000 youth under age 20 years are diagnosed with Type 1 diabetes (T1D) annually [1]. For individuals with T1D, maintaining optimal current and future health outcomes requires adherence to a complex daily regimen with multiple behavioral demands such as monitoring blood glucose, counting carbohydrates, and dosing insulin at appropriate times throughout the day [2]. Additional factors, such as illness or physical activity, may require additional adjustments to the frequency, timing, and calculations associated with insulin self-management.

Substantial empirical evidence links adherence to one’s T1D regimen to favorable glycemic control [3]. Glycemic control is most commonly assessed via the 2-3 month average of an individual’s glucose levels assessed with the measurement of hemoglobin A1C (A1C). Maintaining glycemic control is in turn predictive of reduced long-term risks for retinopathy, cardiovascular disease, and kidney disease [1]. Keeping daily blood glucose values within the target range also reduces short-term risks for hypoglycemia, hyperglycemia, depression, and other unfavorable outcomes [1,4,5]. However, research indicates that many individuals with T1D, and especially adolescents, do not maintain an optimal level of glycemic control, as defined by the American Diabetes Association [6]. This is related to hormonal changes during puberty and to declines in adherence during adolescence [7-9].

As such, numerous experts have cited a need for increased attention and efforts to boost adolescent adherence [3,8]. Several authors have cited opportunities to use commonly available technologies in these intervention efforts [10-13], particularly given the high penetration of mobile technologies among US adolescents [14,15]. Adolescents with T1D and their parents may already be turning to technology to support diabetes self-management [16]. In fact, there has been a dramatic rise in digital platforms and programs aimed at assisting diabetes self-management, such as mobile phone apps, websites, and groups on social networking sites (such as Facebook and Twitter). For example, as of February 2015, entering “diabetes” into the iTunes app store yielded over 1100 unique results. Yet, despite the number of apps, the evidence base for their adoption and efficacy is lacking [17].

Many technologies offer readily available means for acquiring information on demand, communicating with parents and others, and obtaining feedback on blood glucose patterns [16]. Parents’ use of technologies for diabetes care or information may also be important, as research indicates that parents’ technology use is often predictive of adolescents’ parallel behaviors [18], and that parental monitoring and involvement in youth T1D care predicts adolescents’ diabetes self-management and glycemic control [19-22]. Given the penetration of a variety of digital communication channels and technologies, it is reasonable to expect that adoption of common technologies for diabetes self-management is prevalent among adolescents with T1D and their families.

However, despite increased access to digital resources, little is known regarding how many adolescents with diabetes make use of diabetes-specific technologies or what relationships exist between use and adolescents’ self-management behaviors and glycemic control. Given the lack of information on the prevalence or role of technology use in pediatric T1D, it is especially important to look at patterns associated with individual technologies, as they may be used by different subsets of adolescents with T1D and/or be uniquely linked to self-management behavior or glycemic control.

This study examined the use of 5 commonly available technologies for diabetes among adolescents with T1D and their parents. The main aim of the study was to identify the proportions of adolescents and parents who adopt each technology and the number of different technologies they use for diabetes. In addition, analyses investigated the reasons adolescents report for using or not using various technologies for diabetes, and whether demographic, parental, and clinical factors correlate with their use of each technology. A final aim was to identify whether the different types and/or number of technologies that adolescents use for diabetes were related to self-management and glycemic control.

## Methods

### Sample and Procedures

Recruitment took place within a large regional pediatric diabetes clinic in an academic medical center and through the Children with Diabetes community website. Children with Diabetes (CWD) is an organization that provides Web-based and face-to-face support and education for young people living with T1D and their parents. Adolescents were eligible for the study if they had been diagnosed with T1D for at least 6 months, were between 12-17 years of age, had no cognitive or sensory impairments that would prevent completing a questionnaire, and had access to the Internet.

Potential pediatric diabetes clinic participants were identified through electronic medical records. Parents of all potentially eligible adolescents seen in the diabetes clinic were identified through medical records and initially contacted through a letter sent home inviting participation for themselves and their child (n=485). Of those, 166 (34.2%) completed the survey. A Web address for consent and a Web-based questionnaire was provided in the letter. For CWD, a banner ad and a Web link were provided on the CWD website. Parents who used the hypertext link (n=57) and completed questionnaires through the CWD website received a follow-up telephone call from research staff to confirm their child’s diagnosis of diabetes. Data from families that could not be contacted by telephone to confirm the diagnosis were excluded from analyses (n=21). For both recruitment settings, once a parent had completed Web-based consent the questionnaire URL was sent to their child in an email or text message. Parents completed questions regarding demographics, child clinical information, and technology access and use. If interested, the adolescent was then able to assent and completed the Web-based questionnaire. Study data were collected using the Research Electronic Data Capture (REDCap) [23]. The Web-based survey was closed once the study met recruitment goals (n=174).

### Measures

#### Survey Development

Standardized measures were used whenever possible, but were not available for assessing adolescents’ and parents’ use of technology for diabetes. In order to address this area, a multidisciplinary team of diabetes professionals (pediatric psychologist, nurse practitioner, pediatric endocrinologist) constructed applicable items. All items were pilot tested with 5 parents and 5 adolescents with T1D to confirm readability, comprehension, and content coverage.

#### Demographic and Clinical Characteristics

Parents reported their number of years of education and marital status. Parents also reported adolescents’ age, gender, and race/ethnicity. Median household income was obtained using patient addresses and data from the US Census American Community Survey [24]. Household income was a continuous variable (values ranged from $12,500 to $236,000). Each parent reported the age at which the adolescent had been diagnosed with T1D and whether s/he used an insulin pump.

#### Diabetes Self-Management

The Self-Care Inventory-Revised (SCI-R) was used to measure adolescents’ self-reported diabetes management behavior. The 15-item SCI-R questionnaire has demonstrated internal consistency and predictive validity for A1C [25-27]. Content focuses on a variety of daily self-care tasks such as blood glucose monitoring, insulin dosing, and food choices, as well as behaviors, such as wearing a diabetes bracelet. Participants rated how frequently they performed self-management tasks in the past 1-2 months on a 5-point Likert scale (from 1 = “Never” to 5 = “Always”). Items were averaged and converted to a 0-100 point scale, where higher values represented better self-care [25]. Cronbach alpha was 0.78 in this study.

#### Access to Technologies

Each parent completed items related to the adolescent’s access to technologies in the home (desktop or laptop computer, tablet device), their own mobile phone, and their child’s mobile phone.

#### Adolescent Technology Use

Adolescents completed items that assessed their use of social networking (in general and for diabetes; eg, Facebook, Twitter), diabetes-focused websites, mobile diabetes apps, text messaging communication about diabetes, and software with a blood glucose meter or insulin pump. The survey first asked whether adolescents used the technology at all for diabetes (yes/no), and then a follow-up item asked users to indicate their frequency of use of that technology for diabetes in the past 3 months (from “not at all” to “everyday”).

Technology use for diabetes was examined both as the number of technologies used and frequency of use. For the number of technologies used, we created a summative adolescent index for use of technologies for diabetes using 5 items with dichotomous response options (yes/no). The items were the following: use of social networking for diabetes, visiting diabetes websites, use of mobile diabetes apps, text messaging for diabetes, and use of software associated with the insulin pump and/or glucometer. Each yes was scored 1; the possible score range for the index was 0-5.

#### Parent Technology Use

Parents reported their use of common technologies in general and in the context of their children’s diabetes care (with the exception of glucometer/insulin pump software). With regards to Web-based social networking and apps, parents were first asked if they used these at all. Parents who reported use of the technology were then asked whether they used the respective technology (yes/no) for their children’s diabetes care (eg, “Do you use apps focused on diabetes?”). Parents were also asked whether they “visit websites that focus on diabetes” (yes/no), and, within the past 3 months, whether “[my child] texts his/her blood glucose numbers [to me].” A summative index of parents’ use of technologies for diabetes was created out of these 4 dichotomous items (ie, index ranges from 0 to 4).

#### Reasons for Using or Not Using Technology

For 4 of the technologies, adolescents who reported using each technology responded to 6 items regarding possible reasons for that use. On a 5-point scale from “strongly disagree” to “strongly agree,” participants indicated whether each technology (1) “helps me to better understand how to take care of diabetes”; (2) “helps me to keep my blood sugar numbers in the target range”; (3) “helps me to solve problems related to diabetes”; (4) “helps me share specific information, like blood glucose values, with other people”; (5) “lets me help other people with diabetes”; and (6) “helps me to feel better about living with diabetes.” These questions were not asked with regards to text messaging, which is markedly different from the other technologies, because questions regarding text messaging as “helping” with blood glucose values were confusing to adolescents. Dichotomous variables indicating agreement (yes or no) were created by coding responses of 4 (agree) and 5 (strongly agree) as 1, and responses of 1 (strongly disagree), 2 (disagree), and 3 (neutral) as 0.

Adolescents were asked how they used text messaging for diabetes via a single “select all” item. Six possible uses as well as an open-text “other” option were provided. The 6 options were (1) text a parent or family member blood glucose levels, (2) get diabetes reminders from a family member, (3) text about diabetes to friends, 4) text a member of my diabetes care team, (5) get supportive messages from family or friends, and (6) get automated messages about diabetes.

If adolescents reported not using a technology for diabetes, they were asked an open-ended question regarding why: “What would you say is the main reason you haven't used ‘X’ in the last 3 months for diabetes?” Short answers were categorized by 1 author and 1 research assistant. No discrepancies in categorizing responses were noted. Response rates for these open-text questions do not reflect the total number of adolescents who reported not using a technology because responses to open-text questions were not required in the survey system.

#### Glycemic Control

Medical records were reviewed to obtain A1C values within 3 months before or after completing the Web-based questionnaire. Thus, A1C data were available for participants from the diabetes clinic, but not for participants recruited through CWD. Adolescent A1C was measured with the DCA Vantage Analyzer (Range: 2.5% to 14%, Siemens Healthcare Diagnostics Inc.).

### Analytic Approach

Descriptive analyses were used to examine the distribution frequencies of use of each technology for diabetes, to examine frequencies of diabetes technology index scores, and to examine the distribution of adolescents’ endorsements of possible reasons for using each technology.

This study was exploratory in nature. With no prior effect size estimates available for study variables, we attempted to detect small correlational effect sizes. The sample size needed to detect a correlation coefficient of .20 was calculated as n=153 with Type I error rate .05 and power at .80.

Logistic regression analyses tested associations between demographic, clinical, and parent variables, and use or nonuse of each of the 5 technologies for diabetes. To determine relationships between demographic, clinical, and parent technology-use variables and adolescents’ scores on the diabetes technology index, multiple linear regression was used. All independent variables were entered simultaneously in these logistic and linear regression analyses.

Finally, multiple linear regression models were constructed examining relationships between use of each type of technology and adolescents’ self-management behavior (SCI-R score), and adolescents’ most recent A1C value, respectively. Six regression models were constructed for each of the 2 dependent variables (SCI-R and A1C). This was done to reduce multicollinearity threats and examine relationships between the individual technologies with each dependent variable. Each model contained the demographic, clinical, and parent technology covariates that were significantly related to either dependent variable. In each respective model, adolescent technology-use variables were entered individually as independent variables, without the other technology-use variables. For example, the first model contained a dummy variable representing whether adolescents use social networking for diabetes (yes = 1). All statistical analyses were completed using SPSS version 22.

## Results

### Sample Characteristics

Means for demographic and clinical variables are in Table 1. Values are provided for the entire sample and for 2 subsamples recruited in different settings. The participants recruited through the CWD website had a broad geographical distribution with no particular geographic emphasis. The subsample of participants recruited through CWD had higher household income, duration of diabetes, and greater insulin pump use. The subsamples (ie, recruited through CWD and the clinic) were combined for subsequent analyses.

**Table 1:** Sample and subsample characteristics.

		Mean (SD) or n (%) Full sample (n=174)	Mean (SD) or n (%) Clinic (n=134)	Mean (SD) or n (%) CWD (n=40)	*P* value
**Parent education, n (%)**					.17
	Less than high school	3 (1.7)	3 (2.2)	0 (0)	
	High school	49 (28.2)	41 (30.6)	8 (20.0)	
	2-year college	24 (13.8)	20 (14.9)	4 (10.0)	
	4-year college	62 (35.6)	41 (30.6)	21 (52.5)	
	Master’s	30 (17.2)	25 (18.7)	5 (12.5)	
	Doctoral or JD/MD	6 (3.4)	4 (3.0)	2 (5.0)	
Household income (thousands of dollars)		65.2 (34.5)	60.6 (27.8)	80.5 (48.4)	.001
Married, n (%)		140 (80.5)	107 (79.9)	33 (82.5)	.48
Adolescent age (years)		14.47 (1.65)	14.52 (1.69)	14.30 (1.52)	.43
Adolescent gender, n (% male)		76 (43.7)	61 (43.7)	15 (43.8)	.99
**Adolescent race, n (%)**					.30
	White	149 (85.6)	113 (84.3)	36 (90.0)	
	African American	17 (9.8)	14 (10.4)	3 (7.5)	
	Asian/Pacific Islander	3 (1.7)	2 (1.5)	1 (2.5)	
	Hispanic	7 (4.0)	7 (5.2)	0 (0)	
Duration of diabetes (years)		5.83 (3.53)	5.47 (3.59)	7.02 (3.01)	.01
Use insulin pump, n (% yes)		108 (62.1)	77 (57.5)	31 (77.5)	.02
Self-management (SCI-R)		3.89 (0.49)	3.88 (0.49)	3.95 (0.46)	.41
Medical record A1C		N/A	9.03 (1.91)	N/A	N/A

### Technology Access and Use

Nearly all adolescents had access to a home laptop or desktop computer (97.7%, 170/174) and mobile phone (94.3%, 164/174), with 11% (18/164) sharing their phone with another family member. Of those with access to a mobile phone, 74.4% (122/164) used a smart phone. More than 60% of adolescents in this sample used social networking (62.6%, 109/174). As shown in Table 2, the technology most commonly used for diabetes among adolescents in this sample was text messaging (52.9%), followed by mobile apps (44.8%), and pump/meter software (43.7%). Adolescents on an insulin pump reported high rates of pump/meter software use (50.9%, 55/108) compared to those who did not use a pump (31.8%, 21/66). The least commonly used technologies for diabetes were social networking (27.6%) and websites (24.7%). Among parents, the most commonly used technology for diabetes were websites (60.9%, 106/174), followed by mobile apps (32.8%, 57/174), text messaging with their adolescent about blood glucose (28.2%, 49/174), and social networking (27.6%, 48/174).

Among adolescents who used social networking for diabetes, the frequency of use reflected a fairly even distribution across the categories (from less than once a month to 4 or more times per week). Adolescents who consulted websites about diabetes tended to do so infrequently, as 65% of diabetes website users reported using these websites once a month or less. Adolescents who used apps, text messaging, and pump/meter software for diabetes tended to do so with greater frequency. Of those adolescents who used a given technology, more than 50% of users reported using the respective technology twice a week or more (see Table 2).

**Table 2:** Percentages of technology use and frequency of use for diabetes among adolescents who reported using a technology more than “not at all.”

		Frequency of use					
	Use at all n (%)	Over one time/month n (%)	One time/month n (%)	Two times/month n (%)	One time/week n (%)	Two to three times/week n (%)	Over four times/week n (%)
Social networking	48 (27.6)	8 (16.7)	8 (16.7)	7 (14.6)	5 (10.4)	12 (25.0)	8 (16.7)
Websites	43 (24.7)	16 (37.2)	12 (27.9)	7 (16.3)	5 (11.6)	1 (2.3)	2 (4.7)
Mobile apps	78 (44.8)	15 (19.2)	5 (6.4)	3 (3.8)	9 (11.5)	9 (11.5)	37 (47.4)
Text messaging	92 (52.9)	4 (4.3)	4 (4.3)	11 (12.0)	16 (17.4)	15 (16.3)	42 (45.7)
Meter/pump software	76 (43.7)	13 (17.1)	14 (18.4)	4 (5.3)	7 (9.2)	1 (1.3)	37 (48.7)

**Figure 1:**
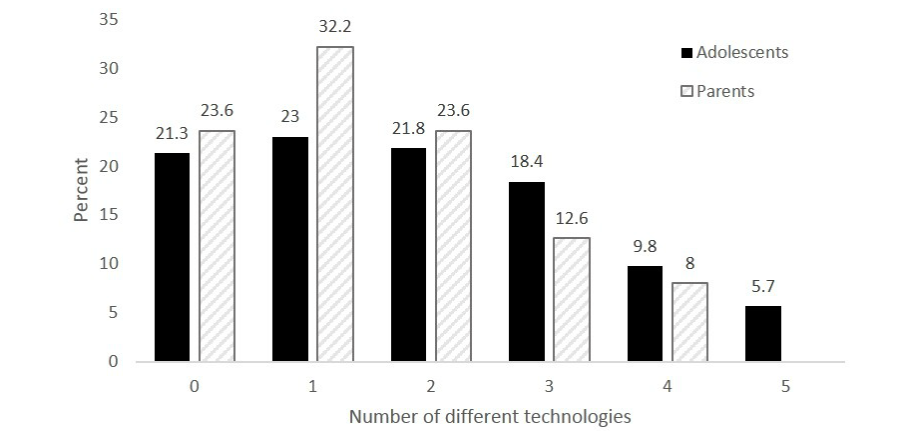
Number of different technologies teens and parents use for diabetes.

The mean score on the adolescent diabetes technology index indicated that on average adolescents used approximately 2 (1.9) of the 5 technologies for diabetes purposes (SD = 1.5, median = 2.0). As shown in Figure 1, there was fairly even distribution across the 5 possible scores. Similarly, parents varied widely in the number of technologies they used in the context of their child’s diabetes care. On average, parents reported using 1.5 of the 4 technologies for diabetes (SD = 1.2).

### Reasons for Using or Not Using Technology

**Figure 2:**
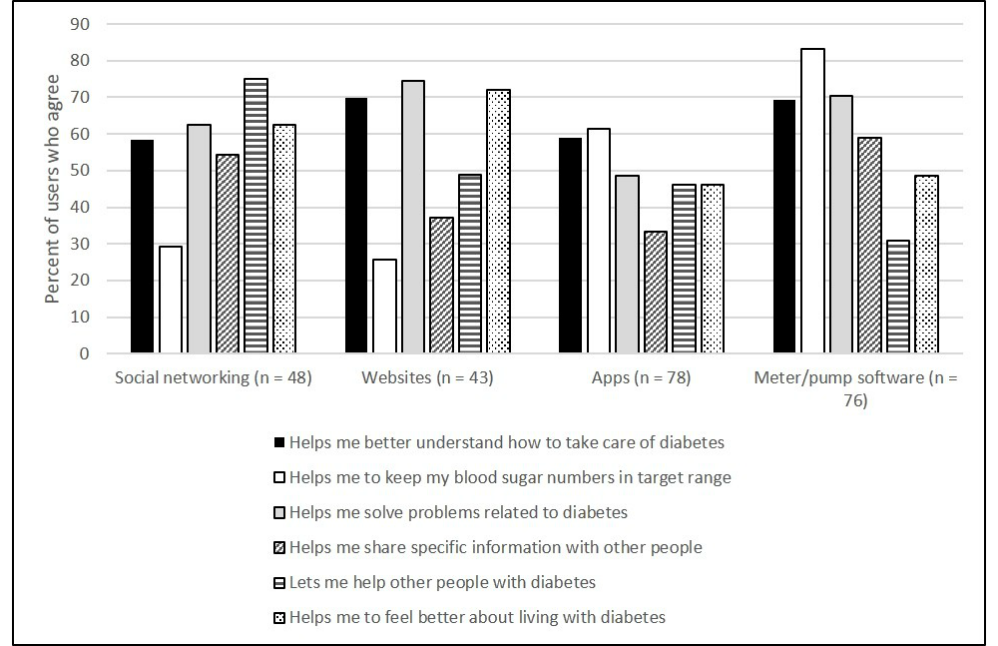
Percent of adolescents who endorsed each reason for using the technology as “agree” or “strongly agree.”

The top reasons for technology use for diabetes varied by technology. The distributions of “agree” or “strongly agree” responses by technology are displayed in Figure 2. Among adolescents who used social networking for diabetes, the most common reason (75.0%, 36/48) for use was that it let them help other people with diabetes. The most common reasons provided for not using social networking for diabetes (n=61 responses) were the following: no need or no problem (21.3%, 13/61), don't want to talk about diabetes (18.0%, 11/61), no time (14.8%, 9/61), no friends with diabetes (11.5%, 7/61), and social networking is not for diabetes (9.8%, 6/61).

Among users of diabetes websites, 74.4% (32/43) agreed that websites helped them solve problems related to diabetes, and 72.1% (31/43) agreed that websites helped them feel better about living with diabetes. If an adolescent did not visit diabetes websites the top reasons noted were the following (n=129 responses): no need (24.8%, 32/129), didn't know of any websites (10.9%, 14/129), used other resources (10.9%, 14/129), or were too busy (8.5%, 11/129).

Adolescents reported that diabetes mobile apps were most commonly used to help keep blood glucose values in range (61.5%, 48/78) and help learn how to take care of diabetes (59.0%, 46/78). If an adolescent did not use mobile diabetes apps they most commonly reported that it was because of the following (n=96 responses): not knowing any apps (20.8%, 20/96), not liking available apps (14.6%, 14/96), no need (14.6%, 14/96), or not wanting to use a diabetes app (6.3%, 6/96).

The largest proportion of adolescents who used their meter/pump software indicated that this technology primarily helped them keep blood sugar numbers in target range (83.3%, 65/78) and helped them solve problems related to diabetes (70.5%, 55/78). The most common reasons for not using the meter or pump software (n=51 responses) included the following: too complicated (19.6%, 10/51), don't know how (15.7%, 8/51), no need (11.7%, 6/51), don't have it (11.7%, 6/51), and unaware it existed (9.8%, 5/51). Reasons for using diabetes-related text messaging included the following: sending parent(s) blood glucose values (91.3%, 84/92), texting a friend about diabetes (34.8%, 32/92), and obtaining general support from family and friends for diabetes (20.7%, 19/92).

### Demographic and Clinical Correlates of Technology Use

Logistic regression models assessed relationships between demographic, clinical, and parent technology-use variables and adolescents’ reported use of each technology for diabetes. For each dependent variable except pump/meter software, parents’ use of the respective technology was entered as an independent variable. The results of these analyses are displayed in Table 3, and show a mix of demographic, clinical, and parent technology-use relationships with adolescents’ use of each technology for diabetes. Adolescent age was positively associated with use of social networking for diabetes (B=0.28, SE=0.14, *P*=.047). Female adolescents were more likely than males to report using diabetes websites (B=0.89, SE=0.39, *P*=.02). With regards to clinical variables, the more recent an adolescent’s diagnosis, the more likely s/he was to use diabetes apps (B=-0.14, SE=0.06, *P*=.01). Adolescents on an insulin pump were more likely to use pump/meter software (B=0.76, SE=0.35, *P*=0.03) and social networking (B=1.74, SE=0.58, *P*=.003). Across models, 2 parent technology-use variables had significant relationships with adolescents’ technology use for diabetes. Parents who used text messaging with their adolescents for diabetes care were more likely to have adolescents who reported using text messaging for diabetes (B=2.30, SE=0.49, *P*<.001), and parents who used apps for diabetes were more likely to have adolescents who also used apps for diabetes (B=1.33, SE=0.37, *P*<.001).

The next analysis examined relationships between demographic, clinical, and parent technology-use variables and the number of different technologies (0-5) adolescents used for diabetes (eg, the adolescent diabetes technology index). In this analysis, parent score on the parent technology for diabetes index was entered as an independent variable, rather than parents’ use of individual technologies. The overall model was significant (*F*_9, 164_ = 4.90, *P*<.001) and predicted 17% of the variance (adjusted *R* ^2^ = 0.17). Adolescents who used insulin pumps reported using more technologies for diabetes on average (B=0.52, SE=0.23, *P*=.03; beta=.17), as did adolescents of parents who used more technologies for diabetes (B=0.44, SE=0.09, *P*<.001; beta=.36).

**Table 3:** Logistic regression models predicting adolescent use of each technology for diabetes.

		Social networking		Diabetes websites		Diabetes apps		Text messaging		Meter/pump software	
		B (SEB)	OR^a^ (CI)	B (SEB)	OR^a^ (CI)	B (SEB)	OR^a^ (CI)	B (SEB)	OR^a^ (CI)	B (SEB)	OR^a^(CI)
**Demographic variables**											
	Parent education	-0.06 (0.19)		0.06 (0.17)		-0.11 (0.15)		0.07 (0.16)		0.02 (0.11)	
	Household income	0.01 (0.01)		-0.001 (0.01)		0.004 (0.01)		0.002 (0.01)		-0.004 (0.01)	
	Parents married	0.03 (0.66)		-0.18 (0.52)		0.01 (0.46)		-0.06 (0.48)		0.33 (0.45)	
	Adolescent age	0.28 (0.14)^b^	1.33 (1.00-1.75)	0.16 (0.12)		0.13 (0.11)		**-**0.19 (0.12)		0.02 (0.11)	
	Adolescent is female	0.76 (0.43)		0.89 (0.39)^b^	2.43 (1.13- 5.22)	0.48 (0.35)		0.12 (0.35)		-0.17 (0.32)	
	Adolescent is non-White or Hispanic	-1.13 (0.86)		-1.48 (0.79)		-0.18 (0.49)		-0.28 (0.53)		-0.40 (0.49)	
**Clinical variables**											
	Duration of diabetes	-0.001 (0.06)		0.001 (0.06)		-0.14 (0.06)^b^	0.87 (0.78-0.97)	0.01 (0.05)		-0.02 (0.05)	
	Uses insulin pump	1.74 (0.58)^c^	5.70 (1.82- 17.9)	0.17 (0.42)		0.08 (0.37)		0.69 (0.39)		0.76 (0.35)^b^	2.14 (1.07- 4.29)
**Parent tech variable**											
	Parent uses respective technology for diabetes	0.48 (0.45)		0.56 (0.43)		1.33 (0.37)^d^	3.78 (1.83- 7.83)	2.30 (0.49)^d^	9.95 (3.85- 25.76)	—	—
Nagelkerke *R* ^2^		0.25		0.11		0.18		0.32		0.06	

^a^OR represents the odds ratio pertaining to adolescents’ use of the respective technology for diabetes (use=1); odds ratios are only included for significant independent variables.

^b^ *P*<.05

^c^ *P*<.01

^d^ *P*<.001

### Technology Use and Self-Management

Multiple linear regression models were constructed to assess individual technology use for diabetes and number of technologies used for diabetes as they related to adolescents’ diabetes self-management (SCI-R) or A1C. For each of the 2 dependent variables, 6 regression models were created (ie, one containing each of the 5 technologies as a predictor, and one with the adolescent technology index). All models contained the demographic, clinical, and parent technology-use covariates found to have a bivariate relationship with either dependent variable, which included household income, adolescent age, adolescent race/ethnicity as something other than non-Hispanic White (dummy variable), and adolescents’ duration of diabetes.

Table 4 shows that adolescents’ use of 3 technologies for diabetes were each related to higher (better) SCI-R scores, including the following: social networking (beta=.18, *P*=.02), websites (beta=.15, *P*=.046), and pump/meter software (beta=.15, *P*=.04). In addition, the greater the number of technologies adolescents reported using for diabetes care the higher their SCI-R score (beta=.23, *P*=.003). In analyses with A1C as the dependent variable, adolescents who reported using diabetes websites tended to have higher A1C values, indicating worse glycemic control (beta=.22, *P*=.01).

**Table 4:** Relationships between technology use for diabetes and adolescent self-management and glycemic control.

	SCI-R^a^		A1C^b^	
	B (SEB)	Beta	B (SEB)	Beta
Household income	0.001 (0.001)	.08	-0.02(0.01)^c^	-.21
Adolescent age	-0.05 (0.02)^c^	-.18	0.20 (0.10)	.18
Adolescent is non-White	-0.18 (0.11)	-.13	1.13 (0.44)^c^	.21
Duration of diabetes	0.01 (0.01)	.08	0.05 (0.05)	.09
Uses social networking	0.19 (0.08)^c^	.18	0.62 (0.38)	.14
Uses diabetes websites	0.17 (0.08)^c^	.15	0.95 (0.35)^d^	.22
Uses diabetes apps	0.12(0.08)	.12	0.26 (0.32)	.07
Uses text messaging	0.10 (0.07)	.11	-0.20 (0.31)	-.05
Uses meter/pump software	0.15 (0.07)^c^	.15	0.19 (0.31)	.05
Adolescent diabetes technology index	0.07 (0.02)^d^	.23	0.17 (0.11)	.13

^a^Self-Care Inventory-Revised; SCI-R model adjusted *R* ^2^ values ranged from 0.05 (text messaging) to 0.08 (technology index).

^b^Glycosylated hemoglobin; A1C adjusted *R* ^2^ values ranged from 0.14 (apps) to 0.18 (websites).

^c^ *P*<.05

^d^ *P*<.01.

## Discussion

### Principal Findings

A primary goal of this study was to provide technology adoption rates for diabetes in adolescents with T1D and their parents. A recent study indicated relatively high use of Internet sources (social networking, websites, and message boards) for diabetes information among parents of children with T1D [28], but to our knowledge this is the first investigation of adolescents’ adoption of commonly available technologies for diabetes. Most adolescents with T1D and their parents reported using at least one commonly available technology for diabetes. Parents and adolescents showed differing patterns of technology use, with teens using text messaging predominantly and parents using diabetes websites. Each of the 5 technologies was used by at least one quarter of adolescents for diabetes purposes. The 3 technologies adopted by the greatest proportions of adolescents for diabetes —text messaging, diabetes apps, and pump/meter software—were also the technologies used most frequently by users (ie, 4 or more times per week). That is, if those technologies were used, they were used frequently. Many participants used at least two technologies for diabetes (56% of adolescents and 44% of parents). However, just under one quarter of the sample did not use any of the technologies for diabetes.

Diabetes-related social networking had a relatively low rate of adoption (~24%). Adolescents reported using social networking primarily because it allowed them to help others with diabetes. Adolescents also commonly reported that social networking helped them to solve problems and feel better about living with diabetes. The reasons provided for not using social networking for diabetes revealed beliefs that one had to disclose a problem in order to bring up diabetes on social networking sites, avoidance of communication with peers about diabetes, the desire to have others with diabetes on the social network, and beliefs that social networking is not intended for or optimized for discussion about diabetes. The integration of popular social networking sites into adolescent chronic health behavior programs will need to address these needs and beliefs. There is little research focused on use of social networking in this population. These results imply that a closed community, safe environment, and minimization of potentially negative consequences of publicly discussing diabetes will likely provide a solid basis for leveraging the potentially positive aspects of social networking such as receiving positive feedback and social support, and sharing diabetes coping and self-management strategies [29-31].

Similarly, adolescents used diabetes websites relatively less than other technologies (~25%). The most common reasons cited for using them included solving diabetes problems, feeling better about living with diabetes, and understanding how to take care of diabetes. When diabetes websites were not used at all, it was typically because adolescents’ believed that they did not need them, did not know of any websites for diabetes, or used other resources. Features common to diabetes websites available to adolescents included a forum for questions and answers, integration with social networking, and news articles or blogs [32]. It is possible that these website features were perceived as largely taken care of through other technologies or resources. Additionally, although websites are available via mobile phones, they may not be viewed or accessed as a mobile resource compared to mobile “apps” with functional components.

Mobile diabetes apps were used by a significant portion (~40%) of the sample. Diabetes apps were used specifically for managing blood sugars with low rates of use for other functions such as communication with others. This is consistent with recent research documenting diabetes app features primarily focused on blood glucose tracking and management [17,33]. The majority of adolescents did not use diabetes apps at all due to not knowing about them, not liking their choices, or not feeling the need to use them. Reasons for not using apps for diabetes point to the need for a mobile intervention evidence base and the integration of adolescents in the design and testing of those tools. These processes will result in a scientific rationale upon which clinicians may recommend mobile apps in routine care and apps that are engaging to adolescents.

Text messaging was used most frequently by adolescents for diabetes compared to the other technologies (~53%). Adolescents primarily used text messaging for communicating blood glucose values with family and for general communication with friends about diabetes. Meter and pump software were also one of the most frequently used technologies (~44%). Not surprisingly, adolescents using an insulin pump used the software most often. One possible reason for the software adoption rate is the bolus wizard function, which may be used up to several times per day. This feature assists in calculating insulin dose but is not required. The reasons for not using software associated with a meter or pump revealed that the majority found the software difficult to use or had not been educated about how or why to use it.

Adoption of the respective technologies was associated with varying demographic, clinical, and parent technology-use variables but did not show strong relationships across technologies. Age, duration of diabetes, and insulin pump use, while related to use of a single technology, did not show robust relationships. Across technologies, use was not associated with socioeconomic status variables. Access to technologies was quite high and overall, socioeconomic status did not appear to impact the frequency of technology use in this sample. However, as with many studies of pediatric T1D, this sample had somewhat reduced variability in income, education, and race. That may have impacted the ability to establish a relationship with those variables.

Adolescents who reported using social networking, websites, and glucometer and/or pump software had better self-reported self-management compared to nonusers. Although their features and purposes vary, more than 60% of adolescents who used each of these 3 technologies for diabetes agreed that these helped them to solve diabetes-related problems. Problem solving skills are consistently related to better self-management in cross-sectional and interventional diabetes research [34-37]. Problem solving value may be a critical determinant of whether or not a given technology is adopted or viewed as helpful for self-management. Next steps for this research will document the specific problems identified and/or solved using these technologies.

Interestingly, use of text messaging and mobile apps, the 2 technologies that did not show relationships with self-management, were the most commonly used technologies for diabetes. While the reasons endorsed for using these technologies could logically relate to improved self-management, it may be that unhelpful modes of use may be counteracting each other or that use is too unstructured and does not translate to improving specific behaviors such as blood glucose monitoring or insulin administration. For example, adolescents reported texting friends almost as frequently as parents. In and of itself, communicating about diabetes more frequently using that technology did not appear to relate to better self-management practices.

The use of more technologies, assessed via the technology index, was positively related to self-management. One possible reason for this is that adolescents who use more technologies in the context of their diabetes care may be more diligent in general about managing their disease. As mentioned above, different technologies may also fulfill different diabetes-related needs, and thus using several technologies could support adolescents in more ways than can be accomplished using a single technology. Alternatively, there may be a general orientation toward technology among some adolescents that facilitates the integration of multiple technologies into everyday diabetes problem solving. Although diabetes is associated with a relatively technology-heavy self-management regimen, no research has identified a general orientation toward or adoption of technology in general as related to better levels of chronic illness health behaviors or outcomes. A significant portion of the sample perceived each technology as useful for diabetes. These results will provide the basis for development of an adolescent health technology adoption model. Variables such as motivations for information seeking, problem solving orientation, perceived usefulness, and ease of use will need exploration as part of the model development [38].

While use of several technologies was associated with more favorable self-management, technology use of any kind did not translate into better glycemic control. Although self-management is critical for glycemic control, relationships with self-management were not robust. Adolescents who reported using websites for diabetes had higher A1C values than those who did not. It is possible that adolescents struggling with glycemic control may be drawn to diabetes websites for tips or self-management aids. Overuse of technologies amongst a small portion of adolescents may have an inherent risk as well, with some research indicating the highest frequency users are not those with the best health behaviors [39]. Further research examining the content of technologies adolescents consult for diabetes-related purposes, subsets of adolescents who overuse technologies, as well as their motivations for consulting those technologies are needed to illuminate mechanisms behind these relationships.

### Limitations

This is the first study to document adoption and reasons for use of technology in adolescents with T1D and relate use of those technologies to self-management and glycemic control. However, several limitations of the study should be noted. Some items related to frequency and purpose of technology use needed to be created as they did not exist in the scientific literature. As we did not conduct an observational study to identify content transmitted and technology features used, the mechanisms of the reported relationships are unknown. While meter/pump software has a relatively limited set of uses, content, and communication capabilities, other technologies are more varied in their features. For example, the lack of a relationship between text messaging for diabetes and self-management may reflect the many potential ways that technology may be used and suggests that the nature and quality of family communication around a child’s T1D may be more important than frequency and mode of communication [40]. Qualitative and longitudinal research is needed to determine the nature of use and identify potential mechanisms underlying relationships with self-management.

These data are cross-sectional, and it is not possible to determine the direction or nature of causality in identified relationships. The technologies may be used routinely regardless of varying diabetes circumstances, proactively to prevent worsened self-management, or reactively to address problems. As there were no standardized measures of diabetes-related use of technologies, we needed to create those items. Finally, given the novel and exploratory nature of the study, the relationships suggested here and derived from multiple significance tests should be confirmed in follow-up research.

### Conclusion

These findings have implications for clinicians and researchers designing interventions targeting adolescent adherence and for parents of adolescents with T1D. The majority of adolescents with T1D have access to digital technologies, and most are using at least one technology as a resource for their diabetes self-management. However, it appears that a significant minority are not oriented at all toward technologies for diabetes self-management. Many did not know about the technologies, did not see their value, did not need them, and/or simply were not interested. This may be related to the lack of an established body of literature linking them to improved outcomes, which in turn may result in little promotion of technologies by clinicians for diabetes care. Even so, not every website or mobile app will have an evidence base and are unlikely to be incorporated into clinical practice. The broad uptake of technologies such as mobile apps will depend on a patient-centered development process, a rigorous evidence base, and social marketing of a few good products. Even then, the use of many technology resources alone does not seem to be strongly tied to better self-management. As Borus (2013, p. 2) contends, “…technology without support to help manage the opportunities it provides is not the answer” [40]. For young people, guidance on their use will be important and integration into a comprehensive set of learning supports and experiences will enhance engagement and efficacy.

## Abbreviations

A1C: Glycosylated hemoglobin
T1D: Type 1 diabetes
